# Immunostimulation of Fibrous Nucleic Acid Nanoparticles Can be Modulated through Aptamer-Based Functional Moieties: Unveiling the Structure–Activity Relationship and Mechanistic Insights

**DOI:** 10.1021/acsami.3c17779

**Published:** 2024-02-12

**Authors:** Laura P. Rebolledo, Weina Ke, Edward Cedrone, Jian Wang, Krishna Majithia, M. Brittany Johnson, Nikolay V. Dokholyan, Marina A. Dobrovolskaia, Kirill A. Afonin

**Affiliations:** † Nanoscale Science Program, Department of Chemistry, University of North Carolina Charlotte, Charlotte, North Carolina 28223, United States; ‡ Nanotechnology Characterization Laboratory, Cancer Research Technology Program, Frederick National Laboratory for Cancer Research Sponsored by the National Cancer Institute, Frederick, Maryland 21701, United States; § Department of Pharmacology, Penn State College of Medicine, Hershey, Pennsylvania 17033, United States; ∥ Department of Biochemistry & Molecular Biology, Department of Biochemistry & Molecular Biology, Penn State College of Medicine, Hershey, Pennsylvania 17033, United States; ⊥ Department of Biological Sciences, University of North Carolina Charlotte, Charlotte, North Carolina 28223, United States

**Keywords:** NANPs, RNA, DNA, nanoparticles, cytokines, cGAS, STING, immunostimulation

## Abstract

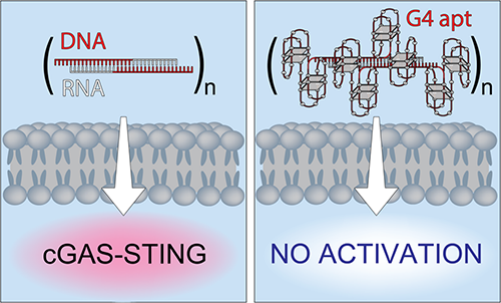

Fibrous nanomaterials containing silica, titanium oxide, and carbon nanotubes are notoriously known for their undesirable inflammatory responses and associated toxicities that have been extensively studied in the environmental and occupational toxicology fields. Biopersistance and inflammation of “hard” nanofibers prevent their broader biomedical applications. To utilize the structural benefits of fibrous nanomaterials for functionalization with moieties of therapeutic significance while preventing undesirable immune responses, researchers employ natural biopolymers—RNA and DNA—to design “soft” and biodegradable nanomaterials with controlled immunorecognition. Nucleic acid nanofibers have been shown to be safe and efficacious in applications that do not require their delivery into the cells such as the regulation of blood coagulation. Previous studies demonstrated that unlike traditional therapeutic nucleic acids (e.g., CpG DNA oligonucleotides) nucleic acid nanoparticles (NANPs), when used without a carrier, are not internalized by the immune cells and, as such, do not induce undesirable cytokine responses. In contrast, intracellular delivery of NANPs results in cytokine responses that are dependent on the physicochemical properties of these nanomaterials. However, the structure–activity relationship of innate immune responses to intracellularly delivered fibrous NANPs is poorly understood. Herein, we employ the intracellular delivery of model RNA/DNA nanofibers functionalized with G-quadruplex-based DNA aptamers to investigate how their structural properties influence cytokine responses. We demonstrate that nanofibers’ scaffolds delivered to the immune cells using lipofectamine induce interferon response via the cGAS-STING signaling pathway activation and that DNA aptamers incorporation shields the fibers from recognition by cGAS and results in a lower interferon response. This structure–activity relationship study expands the current knowledge base to inform future practical applications of intracellularly delivered NANPs as vaccine adjuvants and immunotherapies.

Fibrous materials, such as asbestos, silica and glass fibers, titanium oxide nanobelts, synthetic polymeric fibers, and carbon nanotubes (CNTs), have an established reputation for undesirable inflammation triggers.^1−7^ Inhaled silica-containing fibers cause granulomatous lung inflammation, eventually leading to mesothelioma.^8,9^ Environmental and occupational exposure to CNTs, silica, and titanium oxide-containing fibrous materials was extensively investigated for inflammatory responses recognized at the Department of Human and Health Services level, including the Centers for Disease Control and Prevention.^10−12^ The inability of phagocytic cells to effectively eliminate these materials (i.e., the so-called frustrated phagocytosis), physical damage of cellular membranes and organelles (e.g., lysosomes) by fibrous materials leading to the oxidative stress and NLRP3 inflammasome activation, perturbation of intracellular Ca^2+^ stores, were named among the mechanisms underlying the inflammatory response to these materials and associated with it tissue damage.^4,7,13−17^ Moreover, the presence of glass fibers in therapeutic protein formulations was associated with increased immunogenicity of the protein-based drug products.^18^ These earlier studies demonstrated the negative side of inflammation in response to fibrous nanomaterials, limiting their use in industry and preventing biomedical applications. Concerns about the immunogenicity of therapeutic proteins caused by accidental glass fibers contaminating biotechnology drug products prompted researchers to find ways to avoid nanosized fibers in pharmaceuticals.

Despite this setback setting negative precedence for fibrous nanomaterials, they possess a number of beneficial properties. For instance, fibrous materials offer a large surface area, allowing the delivery of a more significant number of functional moieties than a spherical nanocarrier may offer.^19−23^ Moreover, a nonspherical shape has been suggested to improve the vascular transport of nanomaterials and therapeutic cargo delivery into the interstitial space.^24,25^ To leverage these benefits of fibrous shape while avoiding undesirable inflammatory responses, researchers turned their attention to “soft” and biodegradable materials such as those made of natural RNA and DNA biopolymers. The field of nucleic acid nanoparticles (NANPs) is fast growing and has already demonstrated numerous beneficial, safe, and environmentally friendly applications ranging from therapeutic indications^26−49^ as nanomedicines to the use as structural components of light-emitting diodes, functional photonic devices, and biosensors.^50−54^

Due to their macromolecular polyanionic nature, NANPs are not easily internalized by the immune cells in the absence of a carrier; as such, they are immunoquiescent and do not induce inflammatory cytokine responses when delivered into the bloodstream.^55^ This property enables the extracellular use of NANPs, particularly in cases in which inflammation is undesirable, such as in blood anticoagulation. A recent study demonstrated the successful use of RNA/DNA fibers functionalized with thrombin aptamers and their respective “kill switches” to control blood clotting.^39^ In extensive work combining in vitro human-blood-based assays and in vivo studies in small (i.e., mice) and large (i.e., pigs) animals, the anticoagulant NANPs were effective and well-tolerated. Specifically, these anticoagulant NANPs did not trigger immune stimulation and production of cytokines even when tested at concentrations far exceeding those expected at therapeutic doses.^39^

A series of earlier studies demonstrated that after the complexation with a carrier, NANPs can be specifically delivered to and internalized by the immune cells such as blood monocytes and dendritic cells. Upon such targeted intracellular delivery, NANPs differentially stimulate cytokine production based on their physicochemical properties and architectural parameters.^55,56^ Specifically, NANPs delivered by lipofectamine differentially induce cytokines based on NANPs’ composition (i.e., RNA nanoparticles are more potent cytokine inducers than their DNA counterparts), shape (i.e., globular NANPs show greater immunostimulation than planar and fibrous NANPs while planar NANPs have superior responses when compared to fibrous), size (i.e., larger NANPs are more proinflammatory than their smaller analogs), and functionalization (i.e., functionalized NANPs are more immunostimulatory compared to their nonfunctionalized counterparts).^43,55,56^ These responses depend on endosomal toll-like receptors, particularly TLR7, and culminate with the production of type I and type III interferons.^57^

Moreover, a change in the vehicle used for NANPs’ delivery into immune cells can alter the quality of the immune response as measured by the spectrum of cytokines. Specifically, unlike NANPs delivered using lipofectamine, NANPs with identical physicochemical properties but delivered using polyamidoamine dendrimers induce TNF and IL-1.^58^ Collectively, these studies suggest that carriers influence the route of NANPs entry into the cell and their interaction with different pattern recognition receptors (PRRs), leading to dramatically different innate immune responses. Therefore, NANPs allow for controlling the immune responses both quantitatively (i.e., levels of induced cytokines) and qualitatively (i.e., spectrum of induced cytokines). Thus, the variety of engineered NANPs can be tailored to specific applications: immunoquiescent NANPs are employed for indications not involving immune activation, such as drug delivery, while immunostimulatory NANPs are designed to elicit targeted immune responses for applications intended to activate the immune system such as immunotherapies and vaccines.

All previous studies investigating structure–activity relationships (SAR) between NANPs physicochemical properties and cytokine responses employed globular and planar architectures of NANPs, such as cubes and rings, respectively. While fibrous NANPs were studied, due to their lower immunostimulatory potential as compared to cubes and rings, they were used as controls demonstrating that shape contributes to the immunorecognition of intracellularly delivered NANPs.^40,59−61^ However, the SAR of design elements that may contribute to the immunorecognition of intracellularly delivered fibers has never been investigated thoroughly. Therefore, the present work expands on previously published studies by investigating the role of the engineering design and physicochemical properties of fibrous NANPs in their immunorecognition. This study demonstrates the impact of incorporating aptamers within the RNA/DNA fiber NANPs on the innate immune responses in peripheral blood mononuclear cells (PBMCs) and provides mechanistic insights in reporter cell lines (Figure 1). The fibrous NANPs,^39^ offer unique structures and morphologies influenced by factors such as the total number of attached aptamers, the positioning of these aptamers, and the three-dimensional configurations of each aptamer in NANPs. This is exemplified by two representative aptamer structures, with one consisting of a single G-quadruplex and the other with two symmetric G-quadruplexes. This system presents a generalizable model, as G-quadruplex-based aptamers are well-known and widely used against protein targets in various therapies and diagnostics.^62^ The data from the current study expand the existing knowledge base of NANPs’ immunological properties to inform their future development as vaccine adjuvants and immunotherapies.

**Figure 1:**
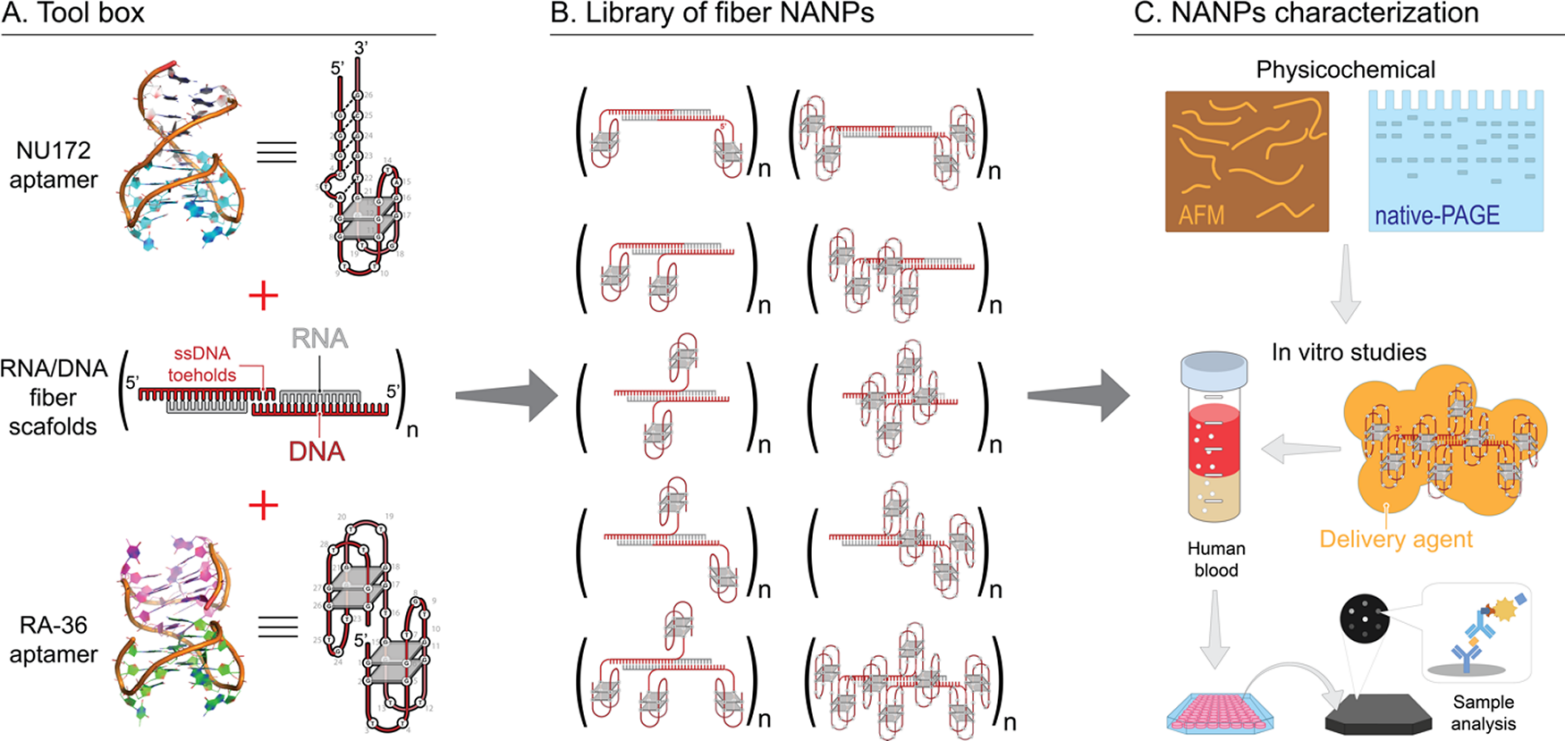
Experimental workflow. (A) Individual components used in this study and their combinations allowed for the production of RNA/DNA fiber NANPs (B) functionalized with different numbers of DNA aptamers. (C) Physicochemical characterization of fiber NANPs and assessment of their immunorecognition in human PBMCs and reporter cell lines.

## Materials and Methods

### Assembly and Characterization of RNA/DNA Fiber NANPs

Assembly and characterization of RNA/DNA fiber NANPs involved the purchase of all individual oligos,^39^ listed in the Supporting Information, from Integrated DNA Technologies, Inc. All RNA/DNA fibers were assembled by combining individual cognate monomers at equimolar concentrations (DNA1:RNA:DNA2:RNA) in hybridization buffer (89 mM Tris, 80 mM Boric Acid (pH 8.3), 2 mM magnesium acetate, 2 mM potassium chloride), heating to 95 °C for 5 min and further incubation at room temperature for 20 min. All assemblies were analyzed at 4 °C on 8% nondenaturing polyacrylamide (19:1) gel electrophoresis (native-PAGE), run for 30 min at 300 V in a hybridization buffer. The Bio-Rad ChemiDoc MP Imager was used to visualize gels stained with ethidium bromide (0.5 μg/mL). After confirming the assembly, RNA/DNA fiber NANPs were kept at 4 °C. For Atomic Force Microscopy (AFM), imaging followed previously developed protocols.^39^

### Isolation of Primary Human Peripheral Blood Mononuclear Cells (PBMCs)^63^

Blood was collected from healthy donor volunteers under the IRB-approved NCI-at-Frederick Protocol OH9-C-N046. The blood was collected in vacutainers containing lithium-heparin as an anticoagulant. The sample was mixed in a 1:1 ratio with phosphate-buffered saline (PBS) at room temperature and layered on top of Ficoll-Paque. Following centrifugation at 900*g* (RCF) with low acceleration and no brake for 30 min at room temperature. The mononuclear layer was collected and washed by adding 3 times the volume of 1X HBSS and centrifugation at 400*g* for 15 min at room temperature. The washing procedure was repeated, and the resulting mononuclear cells were resuspended in a complete RPMI medium (consisting of RPMI 1640 with 10% FBS, 2 mM l-glutamine, 100 U/mL penicillin, and 100 μg/mL streptomycin). Live cells were counted using ViaStain AOPI and used in subsequent experiments.

### PBMCs Treatment with RNA/DNA Fiber NANPs and Controls

To stimulate PBMCs with fiber NANPs for the assessment of cytokine induction, cells were brought up to 1.25 × 10^6^ cells/mL and seeded in 96-well U-bottomed plates with 200 μL per well. RNA/DNA fibers at 1 μM stock solution were complexed to Lipofectamine 2000 (L2K) at a 5:1 v/v ratio. After a 30 min incubation at room temperature, OptiMEM was added, bringing the final concentration of fibers to 50 nM. Afterward, 40 μL of the prepared controls and NANPs were added to PBMC for a final stimulation concentration of 10 nM. As the positive controls, LPS (final 20 ng/mL, Invitrogen), ODN2216 (final 5 μg/mL), and PHA-M (final 10 μg/mL, Sigma) were added to PBMCs. As a negative control, blank media was added to PBMCs. All treatments were added to wells in technical duplicates for each donor. After 20 h of incubation at 37 °C, the plate was spun down at 700*g* for 10 min. 170 μL of supernatant was collected from each well and transferred into a new 96-well plate for analysis of cytokines by multiplexed ELISA (Q-Plex Mouse Cytokine – Inflammation (14-plex) and Q-Plex Human Cytokine – HS Screen (15-plex) from Quansys Biosciences) according to the manufacturer’s instructions. Supernatants from the three positive controls were pooled 1:1:1. Plates were imaged on a Quansys Biosciences Q-View Imager Pro.^63^

### Immune Reporter Cell Lines

The cell lines HEK-Blue hTLR3, HEK-Blue hTLR7, HEK-Blue hTLR9, HEK-Lucia RIG-I, and THP1-Dual cells, were cultivated in accordance with InvivoGen’s protocols under standard conditions of 37 °C and 5% CO_2_. Specifically, ∼50,000 cells per well of HEK-Blue hTLR3, 7, and HEK-Lucia RIG-I cells were seeded onto a flat-bottom 96-well Greiner plate, while HEK-Blue hTLR9 cells were seeded at a density of ∼80,000 cells per well, following the manufacturer’s recommendations. THP1-Dual cells were suspended in each well at a density of ∼100,000 cells per well. Subsequently, all cell lines were transfected on the same day with their respective positive control and RNA/DNA fiber NANPs (final concentration of 10 nM per well). The positive controls for each cell line were prepared as follows: 3 μg/mL 2′,3′-cGAMP, 10 nM RNA cube, 2 μg/mL R848, and 2 μg/mL Poly I:C. Positive control for THP-1 Dual cells included 3 μg/mL 2′,3′-cGAMP, while HEK-Lucia RIG-I cells were treated with RNA cubes, at a final concentration of 10 nM per well. Both positive controls underwent 30 minute incubation at room temperature with L2K prior to transfection onto their respective cells. Additionally, the positive controls used for HEK-Blue hTLR7 included 2 μg/mL of R848, while 2 μg/mL of Poly I:C were used for HEK-Blue hTLR3 and hTLR9. Following transfection of the treatments and positive controls, cells were incubated at 37 °C, 5% CO_2_ for 24 h. Thereafter, cell viability and activation of IRF and SEAP were assessed via QUANTI-Blue assays for all HEK-Blue cells and QUANTI-Luc assays for HEK-Lucia RIG-I and THP1-Dual cells, following the manufacturer’s guidelines. MTS colorimetric assays were performed for all reporter cell lines to assess the post-transfection viability. For assay evaluation, the Tecan Spark plate reader (at an absorbance of 638 nm) was used. To determine normalized fold induction, the samples were evaluated in biological triplicates, averaged, and normalized to the cell-only treatment.

### G140 Inhibition of cGAS

A small molecule, G140 (InvivoGen), was used at 1 μM to inhibit the activity of cGAS-mediated activation of the IRF in THP1-Dual cells. After a 3 h incubation with the G140, cells were treated with RNA/DNA fiber NANPs and 2′,3′-cGAMP, used as the control, incubated for 24 h, and IRF activation was assessed by a QUANTI-Luc 4 Lucia/Gaussia assay.

### siRNA Knockdown of cGAS

24 h prior to the transfections with RNA/DNA fiber NANPs, THP1-Dual cells were transfected with 10 nM siRNA targeting cGAS (ThermoFisher Scientific, assay identification number s41746) using RNAiMAX (ThermoFisher Scientific). Cells were incubated at 37 °C, 5% CO_2_ for 24 h before IRF activation assessment via a QUANTI-Luc assay. In parallel, cell lysates and supernatants were collected for analysis at 24 h post-transfection with RNA/DNA fiber NANPs. Immunoblot analysis for total cGAS protein was conducted to confirm the siRNA-mediated knockdown. Blots were incubated with a rabbit polyclonal antibody against human cGAS (Cell Signaling, cat no. 15102S; 1:1000) overnight at 4 °C. Blots were then washed and incubated in the presence of horseradish peroxidase (HRP)-conjugated secondary antirabbit antibody. Bound antibody was detected with a SuperSignal High Sensitivity ECL kit (ThermoFisher Scientific). Immunoblots were reprobed with a human monoclonal antibody against β-actin (Abcam, cat. no. 49900; 0.13 μg/mL) to assess total protein loading. ImageLab software (BioRad) was used for densitometric analysis.

### Preparation of RNA Cubes and DNA Duplexes for Control Experiments

The RNA cubes were used as a positive control for HEK-Lucia RIG-I cells at a final concentration of 10 nM. Six-stranded RNA cubes were assembled using previous protocols.^49,55,64^ Briefly, six transcribed RNA strands were mixed at an equimolar ratio in endotoxin-free water, heated to 95 °C for 2 min, snap-cooled to 45 °C for 2 min, and 5X assembly buffer, at 20% of the final volume, was added, followed by additional incubation for 20 min at 45 °C. DNA duplexes of various lengths were prepared by mixing complementary strands at equimolar concentrations in endotoxin-free water, and then the samples were heated to 95 °C for 2 min prior to adding 5X hybridization buffer at 20% of the final volume. Lastly, the samples were incubated at room temperature for 20 min and then placed on ice.

### Statistics

All experiments were conducted in a minimum of three biological repeats, unless stated otherwise. Statistical significance was assessed using Student’s *t*-test or ANOVA performed with GraphPad Prism Software. A p-value less than 0.05 was considered statistically significant.

### Molecular Dynamic Simulations

The structures of RNA/DNA fiber NANPs were built through iFoldRNA^65−67^ (the PDB IDs of cGAS and NU172 aptamer are 6CT9 and 6GN7, respectively). The initial designs of human cGAS were built through SWISS-MODEL.^68^ These initial models are then subject to all-atom molecular dynamics simulation through Amber with the Amber^69^ RNA OL3 force field and protein ff14sb force field. The molecules were solvated in an octahedral box containing TIP3P water molecules with a distance of 9 Å maintained between atoms and the box boundary. The system was neutralized, and additional K^+^ and Cl^–^ ions were added to achieve a concentration of approximately 0.3 M. The steepest descent method was employed for 1000 steps, followed by 1000 steps using the conjugate gradient method to perform energy minimization of the entire system. An explicit solvent MD for 500 ns under constant pressure at 1 bar using the isothermal–isobaric (NPT) ensemble was performed with a time step of 2 fs. Finally, the centroid structures obtained from the clusters of the MD simulation trajectories were subject to HDock^70^ docking. The top 100 poses, ranked by HDock scores, were chosen for subsequent hierarchical clustering analyses, employing a threshold of 5 Å for the distance between the centroids of the pose masses.

## Results and Discussion

### Assembly and Characterization of RNA/DNA Fiber NANPs

The original set of RNA/DNA fibers was designed to incorporate multiple copies of antithrombin aptamers (RA-36 and NU172).^39^Table 1 summarizes the architectural parameters, functional characteristics, and spatial differences between all tested NANPs. Each fiber NANP was produced through the self-assembly process of RNA/DNA repeating units. Each repeating unit is made of two RNA and two DNA strands, designed to carry various types of aptamers (e.g., NU172 for constructs 1–5 vs RA-36 for constructs 6–10), which are added through the extension of either 5′- or 3′-ends of DNA oligos. Moreover, different numbers of aptamers per repeating unit are incorporated (e.g., no aptamers for construct 13 vs four aptamers for construct 6). There are also variations in the spacing between aptamers within each unit, with examples like 40 bp for construct 10. The developed design principles allow for the production of RNA/DNA fibers with broad size distributions. This distribution proves advantageous when the constructs are used in vivo, enhancing their overall bioavailability.

**Table 1:**
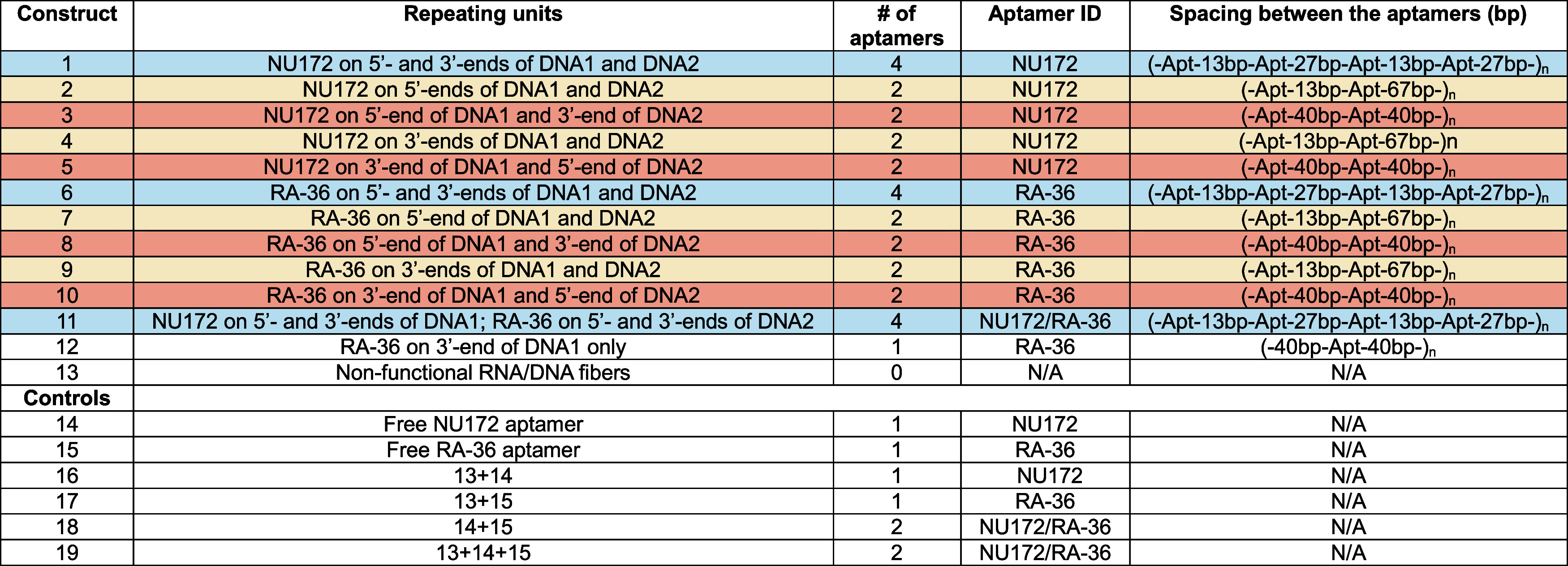
Tested Constructs^a^

a RNA/DNA fibers with identical spacings are color-coded. Samples 14–19 represent the compositions of various tested controls.

**Figure 2:**
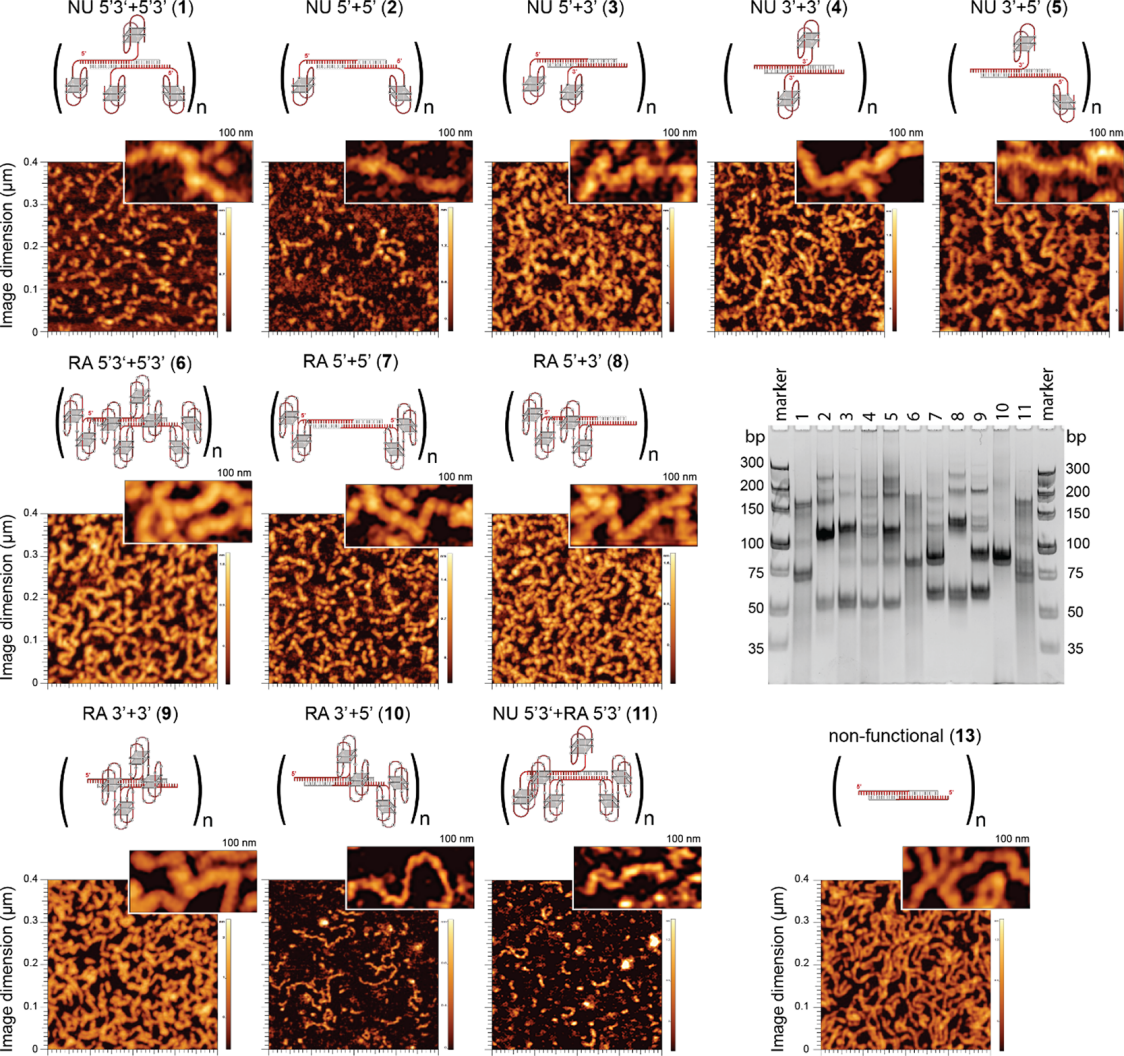
Physicochemical Characterization of RNA/DNA fiber NANPs used in this work. All NANPs’ assemblies are confirmed by native-PAGE and their fiberous structures are verified using AFM (construct numbers are shown in bold).

All assemblies were confirmed by native-PAGE experiments, and the formation of the fibrous structures for all NANPs was verified using AFM (Figures 2 and S1). Consistent with previous work,^39^ the differences in the RNA/DNA fibers’ morphology and size arise mainly from the three-dimensional structures and relative density of incorporated aptamers. Accordingly, fiber NANPs with a higher number of aptamers in their structure show increased flexibility and shorter lengths, as observed, for constructs 10 vs 11. Based on the native-PAGE analysis, all RNA/DNA fibers with a size range of 50–300 bp and beyond were subjected to further testing.

### Cytokine Induction by Intracellularly Delivered Fibers Depends on the Scaffold and Can Be Decreased by Passivating the Fibers Surface with Aptamers

**Figure 3:**
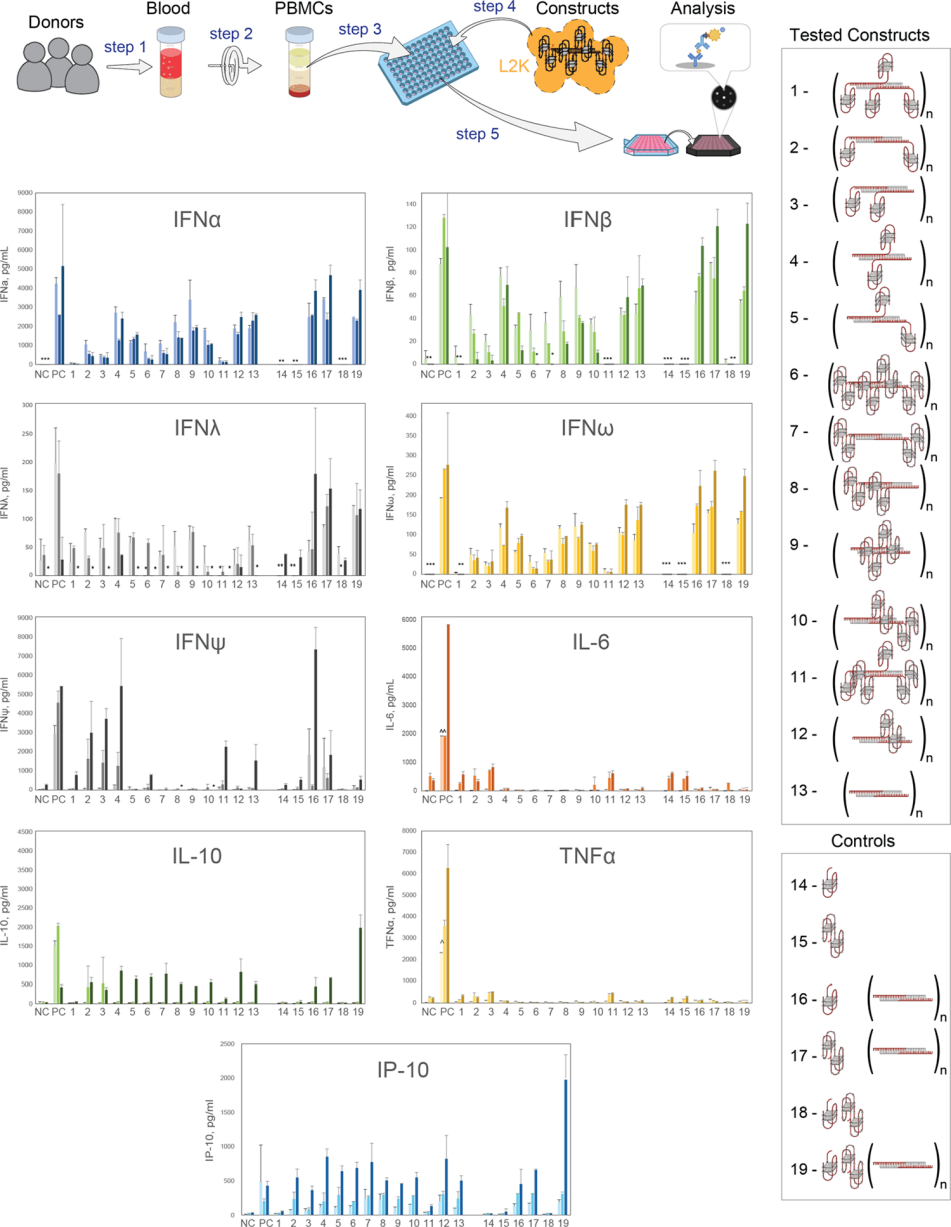
Cytokine secretion by PBMCs. Each bar shows a Mean ± SD (*N* = 4) per donor. Different intensity of color is used to highlight individual donors (*N* = 3). Stars indicate values below the limit of detection. Schematics show the experimental design (top panel) and compositions of the tested samples (right panel).

To investigate the contribution of aptamers and scaffold fibers to the cytokine response in human primary blood cells, we conducted experiments in human PBMCs using fiber NANPs with various engineering designs (Figure 3). Physiologically relevant (i.e., at least 2-fold above the baseline) induction of proinflammatory cytokines, TNF and IL −6, was not observed in cultures exposed to all tested constructs. This finding agreed with the earlier studies demonstrating that interferons, particularly type I (IFNα, IFNβ, IFNω) and type III (IFNλ), are the biomarkers of the lipofectamine-delivered NANPs.^55^ IP-10 induction was detected and indicated the production of type II interferon (IFNγ), which likely occurred as a secondary response to type I interferons.^71^ The observed trends in the magnitude of the interferon response were consistent among detected interferons and IP-10, demonstrating that this biological response is related to the structural properties of the tested samples. For the purpose of brevity, from this point on we will use the cumulative term “cytokines” when referring to interferons and IP-10. Samples 15, 16, and 19 did not induce cytokines above the baseline, i.e., the negative control (PBS-treated PBMCs) (Figure 3). This data indicates that aptamers separately or in combination are not immunostimulatory at the tested concentrations. In contrast, the scaffold DNA/RNA fibers–construct 13 induced significant responses, suggesting that immune cells detect this scaffold as an inflammatory mediator. Induction of cytokines by constructs 1–5 followed the same trend in terms of the cytokine magnitude as that by constructs 6–10, suggesting that the difference in sequences between aptamers NU172 and RA-36 does not contribute to the observed inflammatory response.

Samples 1, 6, and 11 produced responses equivalent to that observed with constructs 2 and 7 and constructs 4 and 9 (Figure 3). Among fibers containing NU172 aptamer, constructs 2 and 3 were less proinflammatory than constructs 4 and 5. Likewise, among fibers containing RA-36 aptamer, constructs 6 and 7 were less potent than constructs 8, 9, and 10. Construct 1, containing 4 units of NU172 aptamer; construct 6, containing 4 units of RA-36 aptamer; and construct 11, containing 4 units of RA-36 and NU172 aptamers, were more immunologically inert – i.e., induced no or very low levels of cytokines–than other tested constructs. These data suggest that the inflammatory response to the RNA/DNA fiber scaffolds can be reduced by adding a higher density of aptamers to the scaffold. Such a design likely interferes with the optimal interaction between the scaffold and innate immune pattern recognition receptors in the cells. Indeed, when aptamers are not attached to the scaffold and simply mixed together, the resulting controls, samples 17, 18, and 20, induce high levels of cytokines. These data further point to the pro-inflammatory nature of the scaffold, which can be masked from immune recognition by attaching aptamers to occupy the scaffold surface.

### Human TLR3, TLR7, TLR9, and RIG-I Are Not Involved in the Immune Recognition of RNA/DNA Fiber NANPs

**Figure 4:**
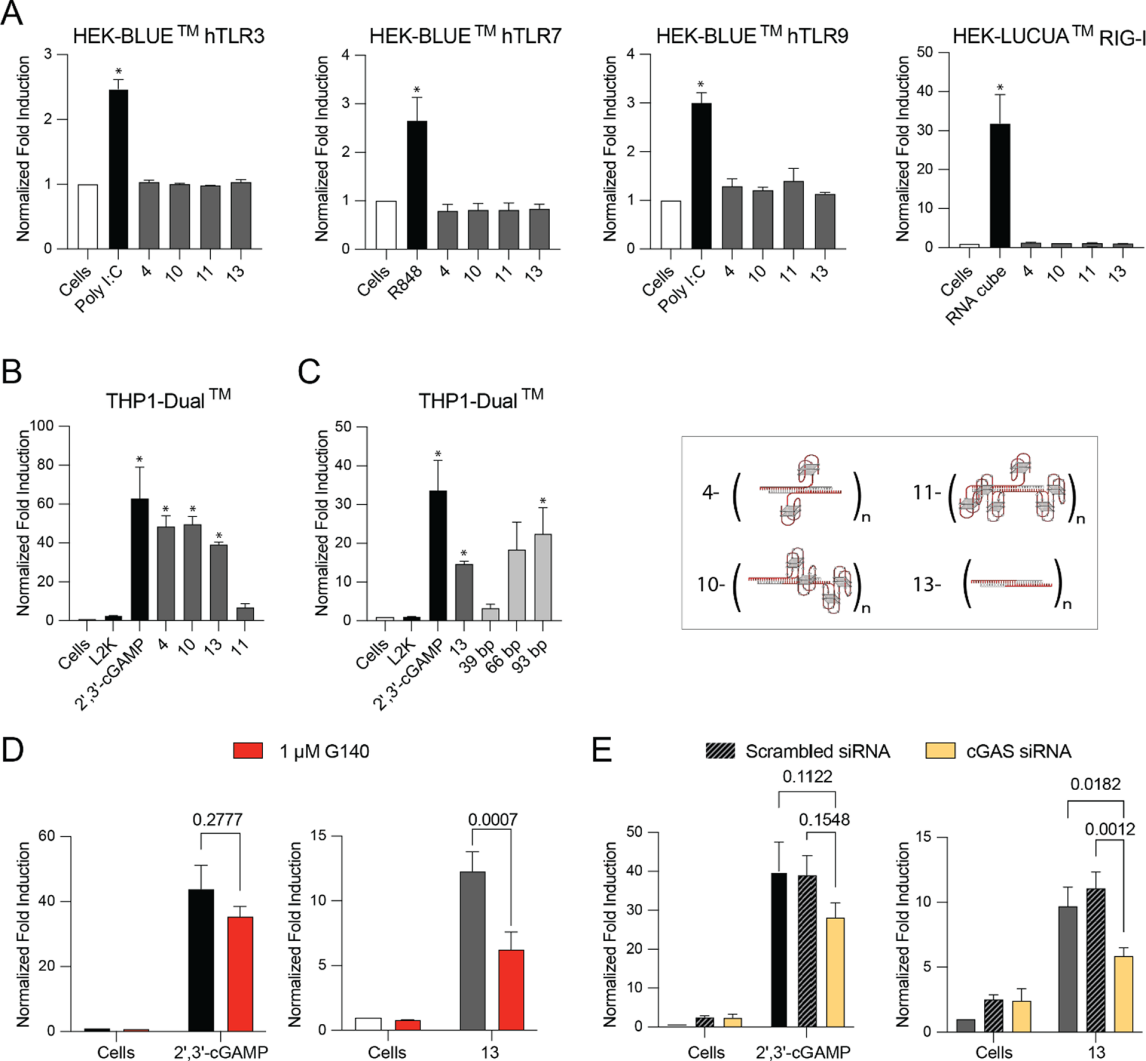
Mechanistic studies of RNA/DNA fiber NANPs immunorecognition. (A) Normalized fold induction of SEAP activation in reporter HEK-Blue hTLR3, HEK-Blue hTLR7, HEK-Blue hTLR9, and HEK-Lucia RIG-I cell lines post-transfection with four RNA/DNA fiber NANPs (B) THP1-Dual cells treated with fiber NANPs. Controls were included to confirm interferon regulatory factor (IRF) activation. (C) Comparison of responses between fiber NANPs and DNA duplexes of different lengths introduced to THP1-Dual cells. In (A–C), asterisks indicate statistically significant differences compared to untreated cells (*N* = 3, Mean ± SEM, Student’s *t*-test). (D) THP1-Dual cells were treated with fiber NANPs and a cGAS inhibitor (G140). Construct 13 was used as a representative example of a cGAS-STING activator. (E) To further explore the mechanism of IRF activation, THP-1 Dual cells were transfected with either scrambled siRNA or siRNA targeting cGAS prior to being treated with 2′,3′-cGAMP or construct 13. In (D–E), corresponding *p*-values are reported (*N* = 3, Mean ± SEM, two-way ANOVA).

To identify the PRRs involved in the recognition of the RNA/DNA fibers, we tested four representative constructs (4, 10, 11, and 13) for their ability to stimulate four different reporter cells: HEK-Blue hTLR3, HEK-Blue hTLR7, HEK-Blue hTLR9, and HEK-Lucia RIG-I (Figure 4A). These reporter cells, engineered to express specific PRRs essential for nucleic acid recognition, are common model systems used in previous studies.^55,56^ Upon testing of the constructs, no significant activation was observed in any hTLR and RIG-I reporter cells, suggesting that these PRRs are not involved in the recognition of RNA/DNA fiber NANPs. Notably, the treatment of all reporter cell lines with the constructs did not result in a significant reduction in cell viability (Figure S3). The selected fibers were additionally tested in THP1-Dual cells, a human monocytic cell line engineered to report the activation of IRF and NF-kB pathways. The analysis revealed IRF activation by constructs 4, 10, and 13 but not by constructs 11 (Figure 4B) in agreement with data obtained using PBMC cultures.

### cGAS Is Involved in the Immune Recognition of RNA/DNA Fibers

To further determine the mechanism of activation, we employed an inhibitor for the cytosolic nucleic acid sensor cGAS that is known to be expressed in THP1-Dual cells (Figure 4B). cGAS has been shown to be a crucial PRR for the recognition of cytosolic dsDNAs as well as RNA/DNA hybrids.^72,73^ Despite the lack of long continuous double-stranded regions in the tested constructs, they do effectively activate cGAS compared to DNA duplexes of varying lengths (Figure 4C). Inhibition of cGAS significantly reduced THP1-Dual cell activation in response to construct 13, suggesting a role for cGAS in RNA/DNA fiber recognition (Figure 4D). Similarly, siRNA-mediated knockdown of cGAS significantly reduced THP1-Dual cell activation in response to construct 13 (Figure 4E). Immunoblot analysis confirmed the knockdown of cGAS protein in these experiments (Figure S2) without affecting cell viability (Figure S3). Collectively, these findings support the hypothesis that cGAS contributes to the recognition of RNA/DNA fiber NANPs, and such recognition can be significantly reduced by the incorporation of multiple copies of aptamers. It is unknown at the moment if the incorporation of other short nucleic acids, such as DNA or RNA oligonucleotides, will have the same effect on the immunostimulatory properties of RNA/DNA fibers as those observed with the G-quadruplex-based DNA aptamers in the current study. Since immunological recognition of traditional RNA and DNA oligonucleotides varies widely based on their sequence, type of the backbone, chemical modifications and other nuances related to the cell type and delivery mode,^74−80^ we hypothesize that the resulting fibers functionalized with such oligonucleotides may have different properties from G-quadruplex-based DNA aptamer functionalized fibers; therefore, future studies with relevant controls specific to each type of such traditional oligonucleotides are warranted.

### Molecular Dynamic Simulations Confirm the cGAS Binding to RNA/DNA Fibers Is Primarily Concentrated in the Double-Stranded Regions

**Figure 5:**
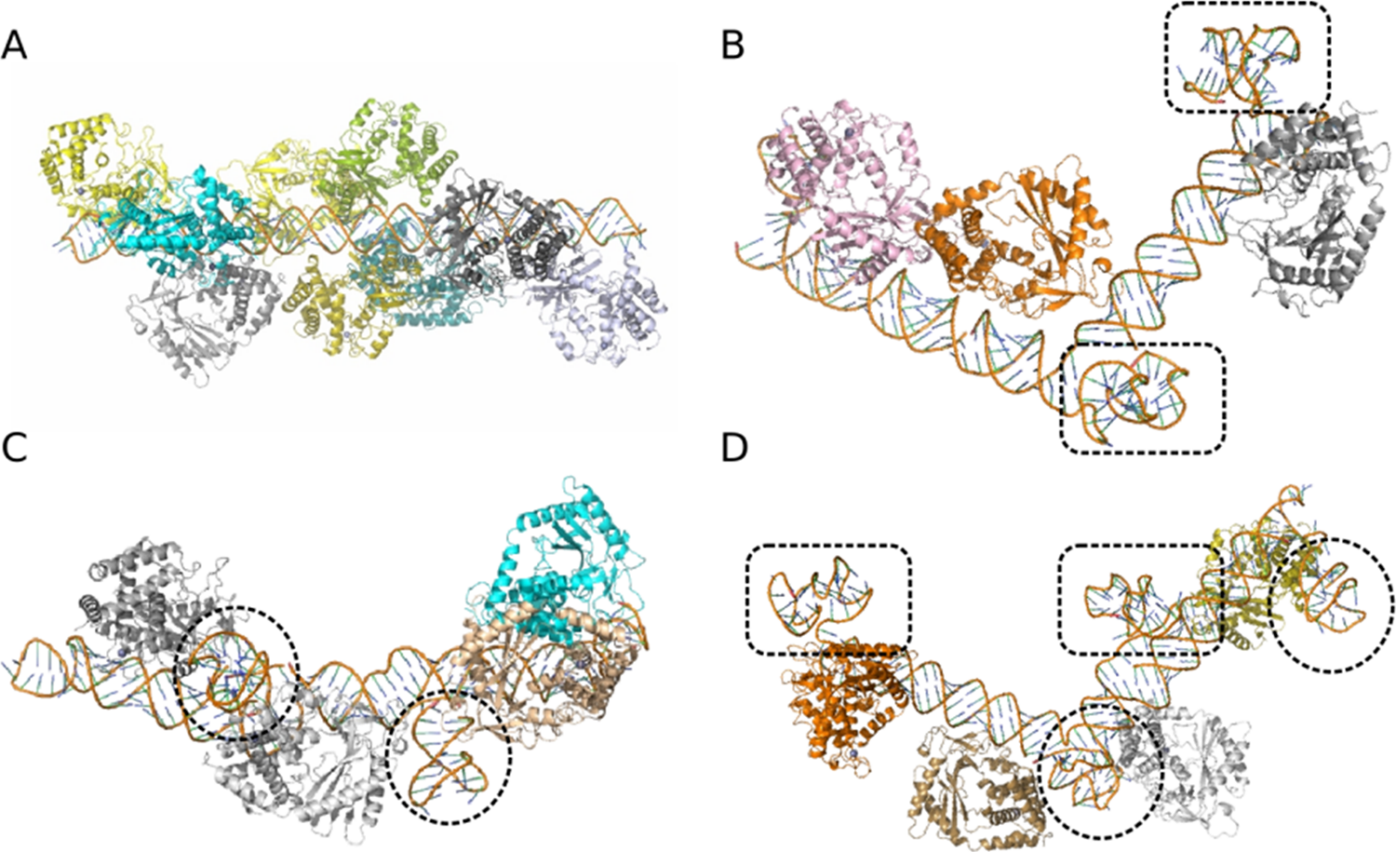
Clusters of the docking pose of cGAS to different fibers. cGAS was docked to constructs 13 (A), 10 (B), 4 (C), and 11 (D), respectively. The RA-36 aptamers are indicated within rounded rectangular frames, while NU172 aptamers are highlighted within circular frames.

To enhance our understanding of this recognition mechanism, the binding between cGAS and fibers was evaluated by using HDock software (Figure 5). When comparing nonfunctionalized RNA/DNA fibers to aptamer functionalized RNA/DNA fibers, the data indicate a difference in their binding affinity with cGAS, as listed in Table 2. While the assessment of binding affinity showed only a slight difference between nonfunctionalized and functionalized fibers, further analysis revealed the divergence in the number of clusters formed by the binding poses. There was a notable disparity in the number of clusters between constructs 13 and cGAS compared to between cGAS and constructs 4, 10, and 11. The decreased number of clusters in the case of aptamer functionalized fibers (constructs 4, 10, and 11) may indicate a distinct structural arrangement or orientation when cGAS interacts with these modified fibers. Notably, cGAS has a known affinity for dsDNA, and our investigation revealed that the binding region of cGAS to these constructs is primarily concentrated in the double-stranded regions (Figure 5). These results collectively suggest a diminished propensity for aptamer functionalized fibers to engage with cGAS. The implications of these structural variances could manifest as variations in recognition via cGAS, downstream signaling events, and activation of innate immune responses, including the production of cytokines and interferons.

**Table 2:** Binding Energy and Clusters of cGAS Docking to Different Fibers

sample #	13	4	10	11
HDock score	–322.85	–319.91	–314.65	–312.43
clusters	11	4	3	4

## Conclusions

Our data indicate that the RNA/DNA fiber scaffolds but not aptamers are the source of the cytokine response following intracellular delivery of the examined constructs into human primary blood cells and reporter cell lines. The results further suggest that the spacing between aptamers and their density on the RNA/DNA fiber scaffold rather than their sequences determine the magnitude of cytokine responses in PBMCs. Unlike globular and planar NANPs, TLRs 3, 7, 9, and RIG-I are not involved in the immunorecognition of RNA/DNA fiber NANPs in the reporter cell lines. cGAS is involved in the immunorecognition of RNA/DNA fibers, and according to the molecular dynamics simulation, this protein interacts with double-stranded regions of the fiber NANPs. This knowledge creates a foundation for employing therapeutic aptamers to stealth-coat otherwise immunostimulatory RNA/DNA fibrous scaffolds to facilitate their immunoquiescent delivery and expand their uses across a spectrum of biomedical applications.

**Figure 6:**
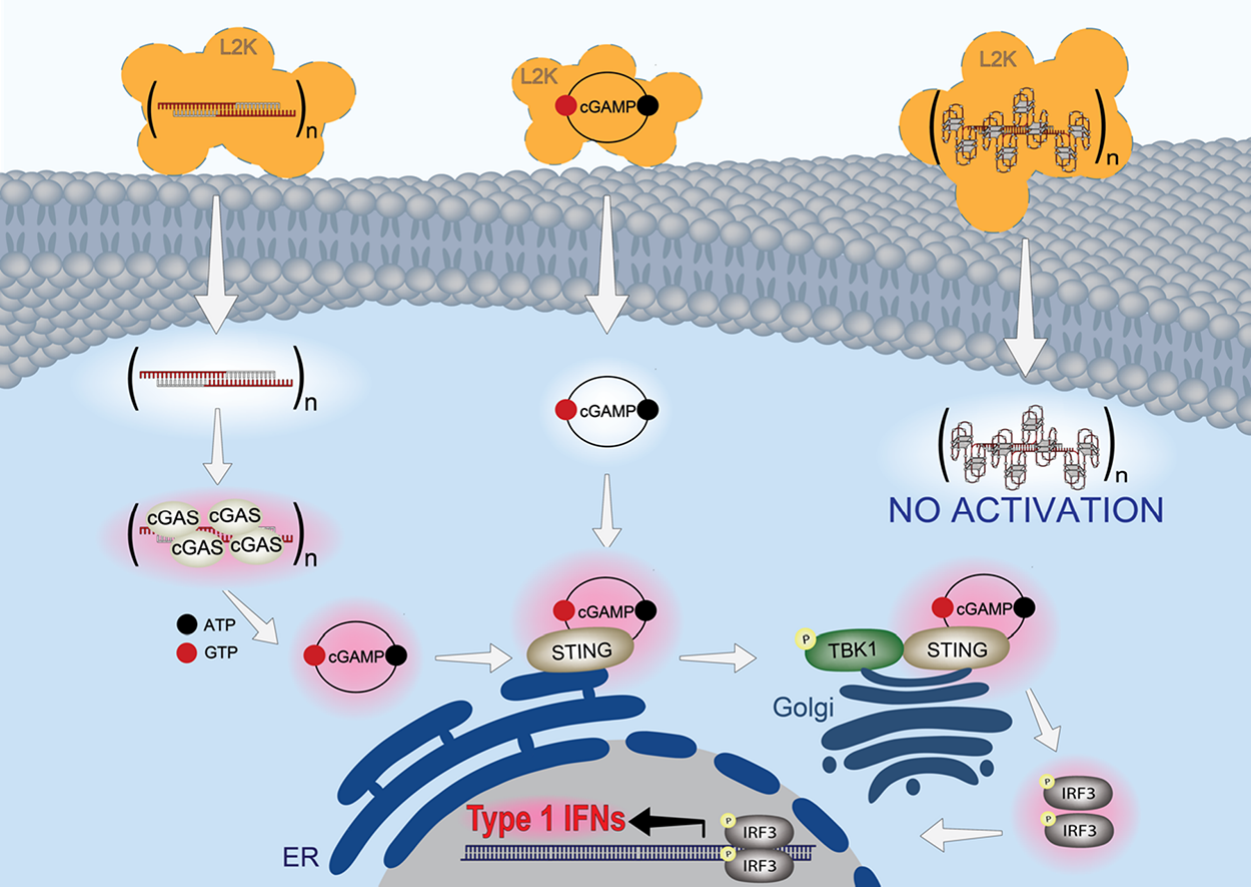
Immunorecognition of RNA/DNA fiber NANPs via the cGAS-STING signaling pathway can be regulated through functionalization with aptamers. The summarizing schematic depicts trafficking and immunostimulation following treatment with nonfunctional fibers and positive control (cGAMP); and the absence of activation in response to fibers functionalized with four aptamers per repeating unit. Orange clouds represent Lipofectamine 2000 (L2K) used for the delivery of all fiber NANPs to the cells.

In this study, we address significant challenges associated with functional RNA/DNA fibers. When introduced into cells as nonfunctionalized scaffolds, these fibers induce the activation of the cGAS-STING signaling pathway, leading to the production of type 1 interferons (Figure 6). The incorporation of DNA aptamers shields the fibers from recognition by cGAS and results in a weaker interferon response.

Collectively, these data expand the current knowledge base of the immunological properties of NANPs and create a foundation to inform the design of fibrous NANPs with desired immunostimulatory profiles. Such knowledge will facilitate the use of aptamers to control the degree of immunostimulation of intracellularly delivered RNA/DNA fibrous scaffolds. This understanding is crucial for their preclinical development as vaccine adjuvants and immunotherapies, thereby expanding the use of these materials across a spectrum of biomedical applications. Additionally, the findings of this study provide valuable insights for the design of future studies, particularly in extending fiber functionalization to include other types of aptamers, such as non-G-quadruplex-based DNA aptamers as well as RNA and DNA oligonucleotides.

## Supporting Information Available

List of all nucleic acid sequences used in this work, siRNA-mediated knockdown of cGAS with representative immunoblots, and cell viability assays for all tested cell lines are shown (PDF)
