# Supplementary material for: Immunostimulation of Fibrous Nucleic Acid Nanoparticles Can be Modulated through Aptamer-Based Functional Moieties: Unveiling the Structure–Activity Relationship and Mechanistic Insights
Source: ACS Appl Mater Interfaces. 2024 Feb 12;16(7):8430–41. doi: 10.1021/acsami.3c17779 (PMC10895590; doi:10.1021/acsami.3c17779)
Supplement: Supplementary file 1 — am3c17779_si_001.pdf [file am3c17779_si_001.pdf]

Supporting Information

Immunostimulation of Fibrous Nucleic Acid Nanoparticles Can be Modulated through Aptamer-Based Functional Moieties: Unveiling the Structure-Activity Relationship and Mechanistic Insights.

Laura P. Rebolledo[1#], Weina Ke[1#], Edward Cedrone[2], Jian Wang[3], Krishna Majithia[5], M. Brittany Johnson[5], Nikolay V. Dokholyan[3,4], Marina A. Dobrovolskaia[2*] and Kirill A. Afonin[1*]

1 - Nanoscale Science Program, Department of Chemistry, University of North Carolina Charlotte, Charlotte, NC 28223, USA
2 - Nanotechnology Characterization Laboratory, Cancer Research Technology Program, Frederick National Laboratory for Cancer Research Sponsored by the National Cancer Institute, Frederick, MD 21701, USA
3 - Department of Pharmacology, Department of Biochemistry & Molecular Biology, Penn State College of Medicine, Hershey, PA, 17033, USA
4 - Department of Biochemistry & Molecular Biology, Department of Biochemistry & Molecular Biology, Penn State College of Medicine, Hershey, PA, 17033, USA
5 - Department of Biological Sciences, University of North Carolina Charlotte, Charlotte, NC 28223, USA

# - equal contributions.
*Author to whom correspondence should be addressed: kafonin@uncc.edu and marina@mail.nih.gov

Keywords: NANPs, RNA, DNA, nanoparticles, cytokines, immunostimulation

Sequences used in this project

RNA/DNA fiber NANPs

Sample #	Compostion	Sequences	
1	Both DNAs (DNA1 and DNA2) in fibers have NU172 aptamers added to their 5` and 3` ends.	DNA 1	5'CGCCTAGGTTGGGTAGGGTGGTGGCGTTTTCCCTTTAGGGAATGACCCTGAAGTTCATCTGCACCACCGAGGGAAATCCCTTTTTCGCCTAGGTTGGGTAGGGTGGTGGCG
		DNA 2	5'CGCCTAGGTTGGGTAGGGTGGTGGCGTTTTTCCCTAAAGGGATGACCCTGAAGTTCATCTGCACCACCGAAGGGATTTCCCTTTTCGCCTAGGTTGGGTAGGGTGGTGGCG
		RNA	5'CGGUGGUGCAGAUGAACUUCAGGGUCA
2	Nu172 Aptamer on 5' sides of fiber DNA1 and DNA2.	DNA 1	5'CGCCTAGGTTGGGTAGGGTGGTGGCGTTTTCCCTTTAGGGAATGACCCTGAAGTTCATCTGCACCACCGAGGGAAATCCCTT
		DNA 2	5'CGCCTAGGTTGGGTAGGGTGGTGGCGTTTTTCCCTAAAGGGATGACCCTGAAGTTCATCTGCACCACCGAAGGGATTTCCCT
		RNA	5'CGGUGGUGCAGAUGAACUUCAGGGUCA
3	Nu172 Aptamer on 5' side of DNA1 and 3' side of DNA2.	DNA 1	5'CGCCTAGGTTGGGTAGGGTGGTGGCGTTTTCCCTTTAGGGAATGACCCTGAAGTTCATCTGCACCACCGAGGGAAATCCCTT
		DNA 2	5'TTCCCTAAAGGGATGACCCTGAAGTTCATCTGCACCACCGAAGGGATTTCCCTTTTCGCCTAGGTTGGGTAGGGTGGTGGCG
		RNA	5'CGGUGGUGCAGAUGAACUUCAGGGUCA
4	Nu172 Aptamer on 3' sides of DNA1 and DNA2.	DNA 1	5'TCCCTTTAGGGAATGACCCTGAAGTTCATCTGCACCACCGAGGGAAATCCCTTTTTCGCCTAGGTTGGGTAGGGTGGTGGCG
		DNA 2	5'TTCCCTAAAGGGATGACCCTGAAGTTCATCTGCACCACCGAAGGGATTTCCCTTTTCGCCTAGGTTGGGTAGGGTGGTGGCG
		RNA	5'CGGUGGUGCAGAUGAACUUCAGGGUCA
5	Nu172 Aptamer on 3' side of DNA1 and 5` side of DNA2.	DNA 1	5'TCCCTTTAGGGAATGACCCTGAAGTTCATCTGCACCACCGAGGGAAATCCCTTTTTCGCCTAGGTTGGGTAGGGTGGTGGCG
		DNA 2	5'CGCCTAGGTTGGGTAGGGTGGTGGCGTTTTTCCCTAAAGGGATGACCCTGAAGTTCATCTGCACCACCGAAGGGATTTCCCT
		RNA	5'CGGUGGUGCAGAUGAACUUCAGGGUCA
6	Both DNAs (DNA1 and DNA2) in fibers have RA36 aptamers added to their 5` and 3` ends.	DNA 1	5'GGTTGGTGTGGTTGGTGGTTGGTGTGGTTGGTTTTCCCTTTAGGGAATGACCCTGAAGTTCATCTGCACCACCGAGGGAAATCCCTTTTTGGTTGGTGTGGTTGGTGGTTGGTGTGGTTGG
		DNA 2	5'GGTTGGTGTGGTTGGTGGTTGGTGTGGTTGGTTTTTCCCTAAAGGGATGACCCTGAAGTTCATCTGCACCACCGAAGGGATTTCCCTTTTGGTTGGTGTGGTTGGTGGTTGGTGTGGTTGG
		RNA	5'CGGUGGUGCAGAUGAACUUCAGGGUCA
7	RA-36 Aptamer on 5' sides of DNA1 and DNA2.	DNA 1	5'GGTTGGTGTGGTTGGTGGTTGGTGTGGTTGGTTTTCCCTTTAGGGAATGACCCTGAAGTTCATCTGCACCACCGAGGGAAATCCCTT
		DNA 2	5'GGTTGGTGTGGTTGGTGGTTGGTGTGGTTGGTTTTTCCCTAAAGGGATGACCCTGAAGTTCATCTGCACCACCGAAGGGATTTCCCT
		RNA	5'CGGUGGUGCAGAUGAACUUCAGGGUCA
8	RA-36 Aptamer on 5' side of DNA1 and 3' side of DNA2.	DNA 1	5'GGTTGGTGTGGTTGGTGGTTGGTGTGGTTGGTTTTCCCTTTAGGGAATGACCCTGAAGTTCATCTGCACCACCGAGGGAAATCCCTT
		DNA 2	5'TTCCCTAAAGGGATGACCCTGAAGTTCATCTGCACCACCGAAGGGATTTCCCTTTTGGTTGGTGTGGTTGGTGGTTGGTGTGGTTGG
		RNA	5'CGGUGGUGCAGAUGAACUUCAGGGUCA
9	RA-36 Aptamer on 3' sides of DNA1 and 2.	DNA 1	5'TCCCTTTAGGGAATGACCCTGAAGTTCATCTGCACCACCGAGGGAAATCCCTTTTTGGTTGGTGTGGTTGGTGGTTGGTGTGGTTGG
		DNA 2	5'TTCCCTAAAGGGATGACCCTGAAGTTCATCTGCACCACCGAAGGGATTTCCCTTTTGGTTGGTGTGGTTGGTGGTTGGTGTGGTTGG
		RNA	5'CGGUGGUGCAGAUGAACUUCAGGGUCA
10	RA-36 Aptamer on 3' side of DNA1 and 5' side of DNA2.	DNA 1	5'TCCCTTTAGGGAATGACCCTGAAGTTCATCTGCACCACCGAGGGAAATCCCTTTTTGGTTGGTGTGGTTGGTGGTTGGTGTGGTTGG
		DNA 2	5'GGTTGGTGTGGTTGGTGGTTGGTGTGGTTGGTTTTTCCCTAAAGGGATGACCCTGAAGTTCATCTGCACCACCGAAGGGATTTCCCT
		RNA	5'CGGUGGUGCAGAUGAACUUCAGGGUCA

11	NU aptamers are on 5' and 3' ends of DNA1, and RA aptamers are on 5' and 3' ends of DNA2	DNA 1	5'CGCCTAGGTTGGGTAGGGTGGTGGCGTTTTCCCTTTAGGGAATGACCCTGAAGTTCATCTGCACCACCGAGGGAAATCCCTTTTTCGCCTAGGTTGGGTAGGGTGGTGGCG
		DNA 2	5'GGTTGGTGTGGTTGGTGGTTGGTGTGGTTGGTTTTTCCCTAAAGGGATGACCCTGAAGTTCATCTGCACCACCGAAGGGATTTCCCTTTTGGTTGGTGTGGTTGGTGGTTGGTGTGGTTG
		RNA	5'CGGUGGUGCAGAUGAACUUCAGGGUCA
12	RA aptamer on 3' end of DNA1 only.	DNA 1	5'TCCCTTTAGGGAATGACCCTGAAGTTCATCTGCACCACCGAGGGAAATCCCTTTTTGGTTGGTGTGGTTGGTGGTTGGTGTGGTTGG
		DNA 2	5'TTCCCTAAAGGGATGACCCTGAAGTTCATCTGCACCACCGAAGGGATTTCCCT
		RNA	5'CGGUGGUGCAGAUGAACUUCAGGGUCA
13	Non-functional fibers	DNA 1	5'TCCCTTTAGGGAATGACCCTGAAGTTCATCTGCACCACCGAGGGAAATCCCTT
		DNA 2	5'TTCCCTAAAGGGATGACCCTGAAGTTCATCTGCACCACCGAAGGGATTTCCCT
		RNA	5'CGGUGGUGCAGAUGAACUUCAGGGUCA
15	NU172 Aptamer	5'CGCCTAGGTTGGGTAGGGTGGTGGCG	
16	RA-36 Aptamer	5'GGTTGGTGTGGTTGGTGGTTGGTGTGGTTGG	

Controls used in experiments with reporter cell lines

DNA duplexes of different lengths

DNA duplexes (bp)		Sequences
39	DNA 1	5'GGAGACCGTGACCGGTGGTGCAGATGAACTTCAGGGTCA
	DNA 2	5'TGACCCTGAAGTTCATCTGCACCACCGGTCACGGTCTCC
66	DNA 1	5'GGAGACCGTGACCGGTGGTGCAGATGAACTTCAGGGTCACGGTGGTGCAGATGAACTTCAGGGTCA
	DNA 2	5'TGACCCTGAAGTTCATCTGCACCACCGTGACCCTGAAGTTCATCTGCACCACCGGTCACGGTCTCC
93	DNA 1	5'GGAGACCGTGACCGGTGGTGCAGATGAACTTCAGGGTCACGGTGGTGCAGATGAACTTCAGGGTCACGGTGGTGCAGATGAACTTCAGGGTCA
	DNA 2	5'TGACCCTGAAGTTCATCTGCACCACCGTGACCCTGAAGTTCATCTGCACCACCGTGACCCTGAAGTTCATCTGCACCACCGGTCACGGTCTCC

RNA cubes

RNA cube - monomers	Sequences
1	5'GGCAACUUUGAUCCCUCGGUUUAGCGCCGGCCUUUUCUCCCACACUUUCACG
2	5'GGGAAAUUUCGUGGUAGGUUUUGUUGCCCGUGUUUCUACGAUUACUUUGGUC
3	5'GGACAUUUUCGAGACAGCAUUUUUUCCCGACCUUUGCGGAUUGUAUUUUAGG
4	5'GGCGCUUUUGACCUUCUGCUUUAUGUCCCCUAUUUCUUAAUGACUUUUGGCC
5	5'GGGAGAUUUAGUCAUUAAGUUUUACAAUCCGCUUUGUAAUCGUAGUUUGUGU
6	5'GGGAUCUUUACCUACCACGUUUUGCUGUCUCGUUUGCAGAAGGUCUUUCCGA

Supporting Figures

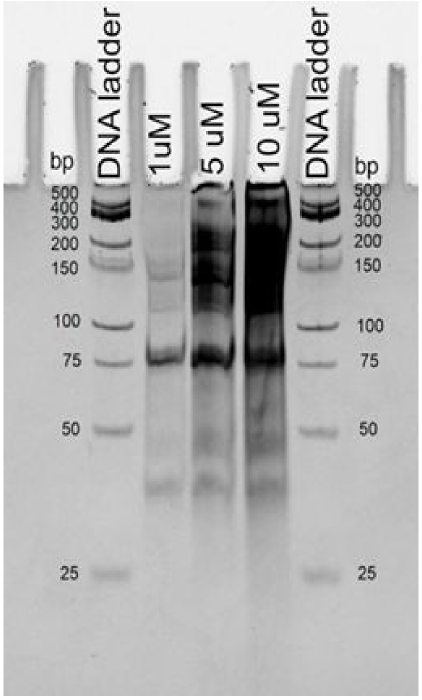

Figure S1: Ethidium bromide total staining native-PAGE verification of non-funcitonal RNA/DNA fibers assembly (sample 13) at different concentrations (1, 5, and 10 μM)

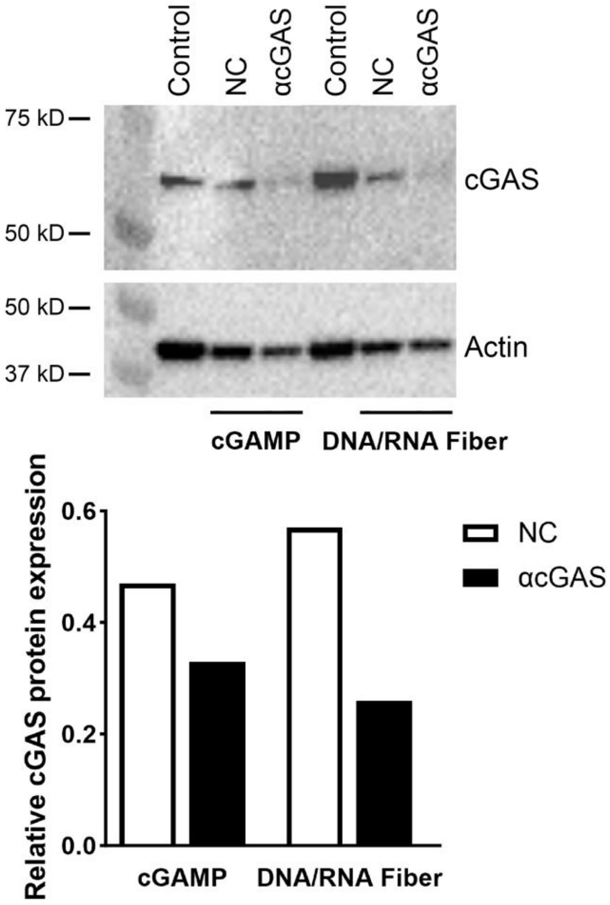

Figure S2: THP-1 dual cells treated with a 10nM concentration of negative control (NC, scrambled siRNA) and siRNA targeting cGAS (αcGAS) for 24h prior to transfection with sample 13 (RNA/DNA fiber) at 10nM. A representative immunoblot analysis demonstrating siRNA-mediated knockdown of cGAS at the predicted size of 62 kD. A representative immunoblot is shown. For the representative immunoblot band intensities for cGAS were determined and normalized to β-actin at the predicted size of 42 kD.

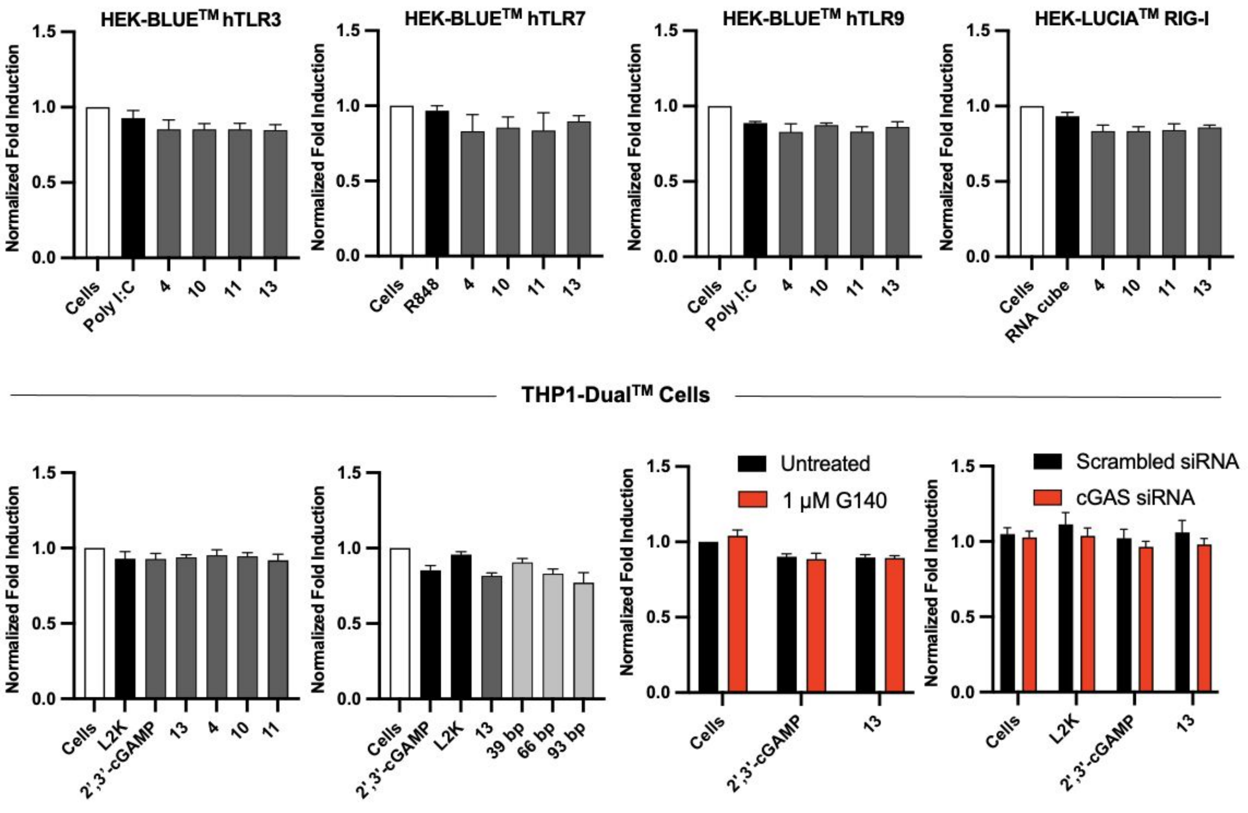

Figure S3: Cell viability of all reporter cell lines treated with RNA/DNA fiber NANPs and relevant controls. Each bar represents the mean of N=3 biological repeats, and error bars denote mean ± SEM.
